# Supplementary figures and images for: Transcriptomic Revelation of Phenolic Compounds Involved in Aluminum Toxicity Responses in Roots of Cunninghamia lanceolata (Lamb.) Hook
Source: Genes (Basel). 2019 Oct 23;10(11):835. doi: 10.3390/genes10110835 (PMC6896160; doi:10.3390/genes10110835)

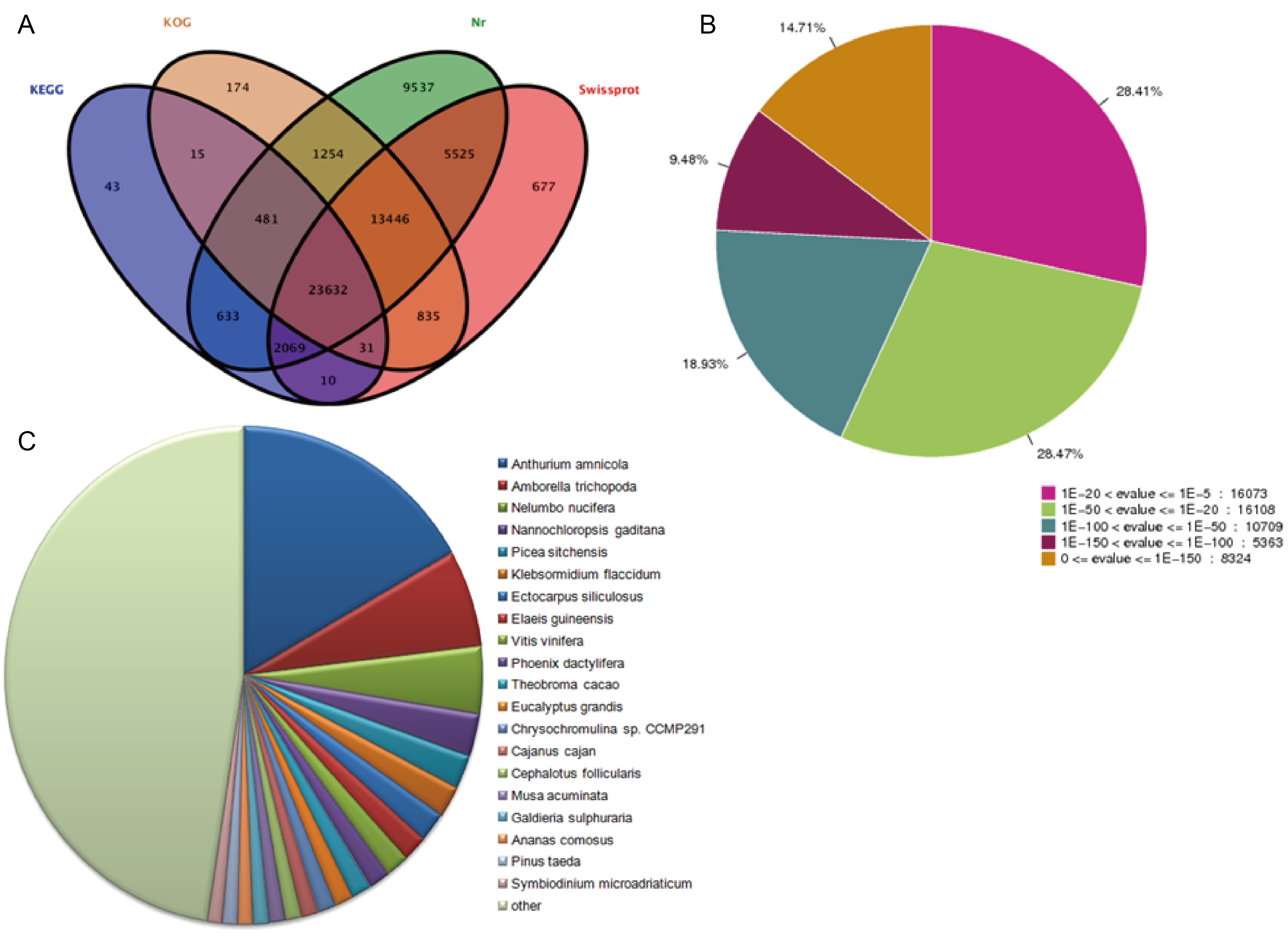

Supplement: Supplementary file 1 [file genes-10-00835-s001.zip › Supplemental files/Figure S1 Characteristics of Chinese fir unigene homologues in four protein sequence databases.png]
